# Mydriasis caused by ESCITALOPRAM: Case report

**DOI:** 10.1192/j.eurpsy.2022.1878

**Published:** 2022-09-01

**Authors:** N. Bouattour, W. Bouattour, N. Messedi, F. Charfeddine, L. Aribi, J. Aloulou

**Affiliations:** Hedi Chaker University Hospital Ain road 0.5 Km, Psychiatry B, Sfax, Tunisia

**Keywords:** mydriasis, escitalopram, Side effects, SSRI

## Abstract

**Introduction:**

Serotonin reuptake inhibitors (SSRIs) are the most commonly prescribed antidepressants thanks to the overall safety and tolerability spectrum. However, they can cause different side effects that not all of them are well identified.

**Objectives:**

We intend to clarify the clinical presentation of mydriasis caused by Escitalopram.

**Methods:**

Reporting the case of a patient suffering a major depressive disorder, that presented a mydriasis after adjusting her antidepressant medication. Then, we conducted a literature review using “PubMed” database and keywords “Mydriasis”, “escitalopram”, “SSRI”,” side effects”.

**Results:**

A 29-year-old female with no past clinical history, presented in May 2021 a severe depression requiring an antidepressant treatment. Under 10 mg per day of escitalopram there was a partial remission of the symptoms, leading to increase the dose by another 10 mg. One month after taking 20 mg/day, she consults before the appointment suffering from a blurry vision and photophobia. Ophthalmologic examination showed a bilateral reactive half-mydriasis, eye pressure was 14 mmHg and fundus examination was normal. Iatrogenic origin of mydriasis was suspected. A gradual interruption of the medication lead to disappearance of the latter. A pharmacological investigation concluded to the suspension of escitalopram and to be vigilant if antidepressant medication would be needed.

**Conclusions:**

Mydriasis is an uncommon side effect caused by SSRI that needs to be kept in mind by clinicians. Therapeutic patient education can help to detect abnormal side effects and treat them if needed.

**Disclosure:**

No significant relationships.

